# Supplementary material for: Picomolar SARS-CoV-2 Neutralization Using Multi-Arm PEG Nanobody Constructs
Source: Biomolecules. 2020 Dec 11;10(12):1661. doi: 10.3390/biom10121661 (PMC7764822; doi:10.3390/biom10121661)
Supplement: Supplementary file 1 [file biomolecules-10-01661-s001.pdf]

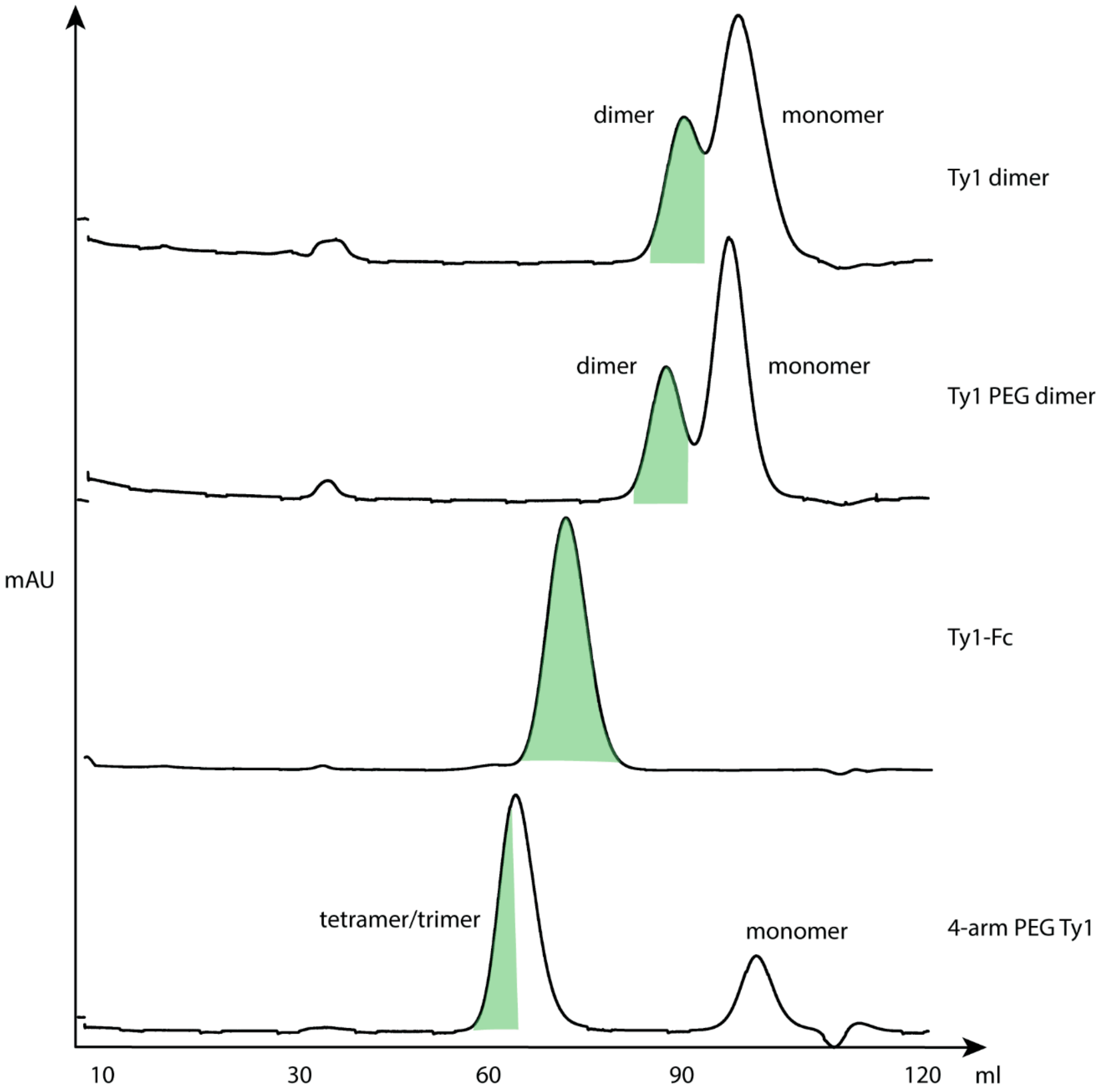

Supplementary Figure S1 Size exclusion elution profiles of the Ty1 dimer, the Ty1 PEG dimer, Ty1-Fc and 4-arm PEG Ty1. All constructs were run on a Superdex S200 16/600 column in 50 mM Tris pH 7.5 and 150 mM NaCl. Fractions indicated in green were collected, concentrated, analyzed by SDS page (Fig. 2B) and used for neutralization assays (Fig. 3). The main peak of the 4-arm PEG Ty1 also contained incompletely reacted, trimeric molecules. To obtain pure 4-arm PEG Ty1, only the first fractions were pooled.

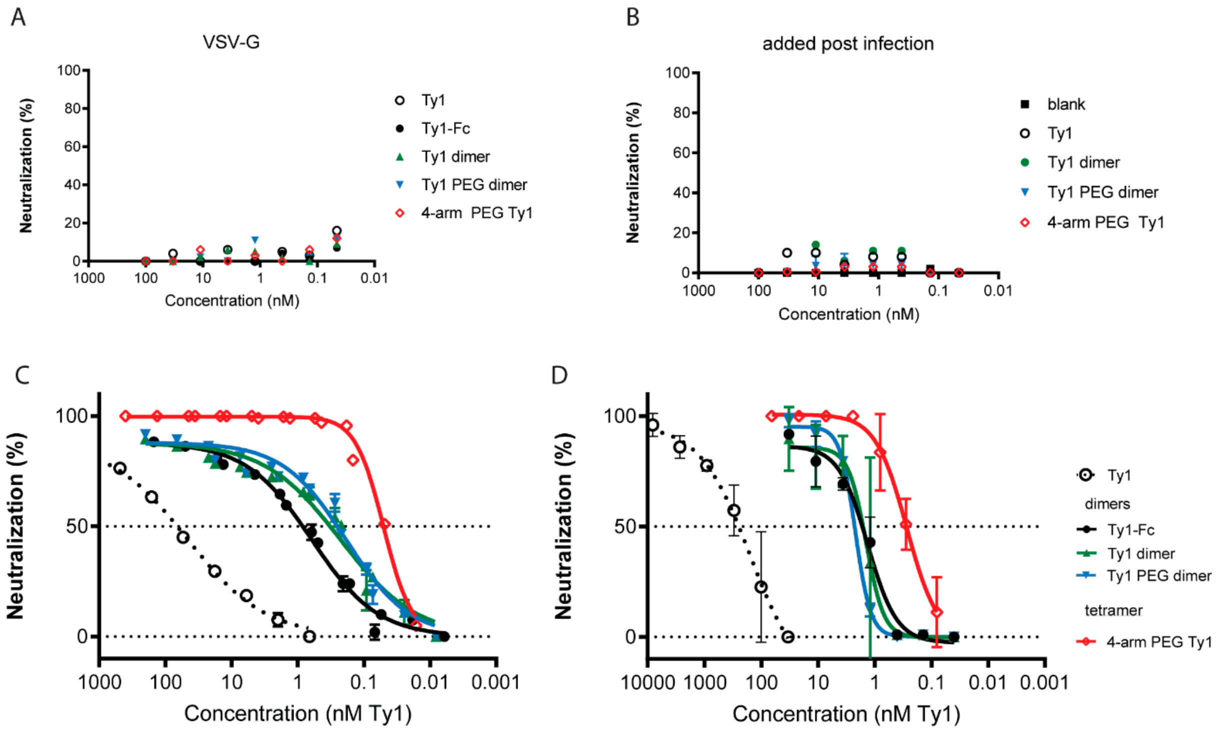

Supplementary Figure S2 SARS-CoV-2 neutralization by multimeric nanobody constructs. (**A**) A dilution series of Ty1 and multimeric nanobody constructs was incubated with VSV-G pseudotyped lentivirus. Neutralization percentage is shown. (**B**) Human ACE2 expressing cells were infected with SARS-CoV-2 pseudotyped lentivirus. After 16 hours, the supernatant was replaced with fresh media including a dilution series of Ty1 and the different constructs. Neutralization percentage, reflected by the reduction in relative light units (RLUs) relative to control wells where no inhibitors were added, is shown. (**C**) SARS-CoV-2 spike pseudotyped lentivirs was incubated with a dilution series of the indicated constructs. Displayed is the same figure as 3A but the concentration is shown in nM of Ty1 subunits. (**D**) Plaque reduction neutralization test using infectious SARS-CoV-2. Same figure as 3B but concentration is shown in nM Ty1.
